# Evaluation of Service-Learning in Project *WeCan* under COVID-19 in a Chinese Context

**DOI:** 10.3390/ijerph18073596

**Published:** 2021-03-30

**Authors:** Hildie Leung, Daniel T. L. Shek, Diya Dou

**Affiliations:** 1 Women’s Leadership Center, Princess Nourah Bint Abdulrahman University, Riyadh 11671, Saudi Arabia; hildiel@gmail.com; 2 Department of Applied Social Sciences, The Hong Kong Polytechnic University, Hong Kong, China; diya.dou@polyu.edu.hk

**Keywords:** COVID-19, service-learning, positive youth development, service leadership, university-corporate-community collaboration

## Abstract

Service-learning is a widely adopted educational pedagogy and philosophy. With the support from the Wharf (Holdings) Limited (Group), service-learning was conducted in the “Project *WeCan*” in Hong Kong. Prior to COVID-19, traditional service-learning was implemented with students learning in the classroom and applying their knowledge and skills to the community through providing direct face-to-face service. With the COVID-19 outbreak in the 2019–20 academic year, school lockdown measures appeared. Students had to learn online and to design and implement service offsite. As the impacts of this rapid shift in paradigm remain unknown, this study examined changes in university students using a pretest–posttest design (*n* = 124) and perceptions of service-learning experience via the subjective outcome evaluation design (*n* = 192) under COVID-19. The authors also investigated service recipients’ (*n* = 56) satisfaction with service activities they participated in during the pandemic. Both objective outcome evaluation and subjective outcome evaluation findings revealed that service providers (university students) and recipients (secondary school students) experienced benefits from the Project. Findings support the benefits of online service-learning in “Project *WeCan*” even during unprecedented times such as COVID-19.

## 1. Introduction

Service-learning (SL) has been a widely-adopted teaching philosophy and pedagogy in the United States for many decades. SL is a credit-bearing experiential learning process where students apply what they have learned from academic studies (i.e., both knowledge and skills) to benefit the community by providing service to those in need [1]. Through SL, community stakeholders benefit from the services provided by students and educational outcomes are attained through critical reflective activities [2]. It is noteworthy that SL has been evaluated mainly through quantitative methodologies [3,4].

### 1.1. Challenges for Service-Learning under COVID-19

At the beginning of 2020, COVID-19 was formally regarded as a pandemic [5]. Countries worldwide aimed to limit the transmission of the virus and flatten the epidemic curve to alleviate the burden on healthcare systems and the resultant macroeconomic crisis especially across China, Russia, India, EU, and leading Emerging Markets [6,7]. Governments and tertiary institutions worldwide launched initiatives and provided guidelines to continue teaching activities while minimizing the spread of the virus [8,9].

While benefits of e-Learning have been well-documented in the literature, most of the studies have been conducted on “traditional” face-to-face courses focused primarily on imparting theoretical knowledge [10,11,12]. In a study investigating the differences in students’ perceived learning experience in practical courses, Elhaty and colleagues concluded that the crisis had negatively impacted students’ acquisition of practical skills as compared to the attainment of theoretical knowledge [13]. This suggests that e-Learning may not be suitable for all types of courses.

COVID-19 has not only exacerbated existing difficulties but also introduced a host of new challenges. Concerns pertinent to the effective delivery of SL during COVID-19 include logistical and technical difficulties, administrative delays, widening implications of the digital divide, the need to explore new communication channels to ensure close contact with community partners, miscommunication among students, issues of trust and open communication due to a loss of shared physical space, and unsatisfactory online curriculum [14,15].

### 1.2. Service-Learning at a Distance

Prior to COVID-19, the increase of technological applications to teaching and learning and the surge of online course offerings have led to the development of online SL (e-SL) [16]. In the case of SL under COVID-19, both teaching and service are online. SL activities can be both direct and indirect. The most prevalent type of SL activities is direct service, where students provide face-to-face service on-site to establish rapport and form relationships and bonds with service recipients. Confronted with the pandemic, indirect services where students can conduct service without physical contact become the obvious alternative. Specifically, these types of services focus on the advocation of social injustice and promulgate consciousness of certain social issues [17]. However, whether students still gain as much as they would have if SL were conducted in person remains unanswered [18,19].

### 1.3. Overview of Project WeCan

The importance of SL to students’ holistic development is now widely accepted across tertiary education institutions in Hong Kong. The number of courses incorporating elements of SL proliferated over the years. Under the Department of Applied Social Sciences at The Hong Kong Polytechnic University (PolyU), two credit-bearing SL courses (APSS2S05 “Promotion of Children and Adolescent Development” and APSS2S09 “Service Leadership through Serving Children and Families with Special Needs”) were launched. These two SL subjects were awarded the Gold Award (Sustainability) and Bronze Award by the QS Reimagine Education Awards, respectively. They were also awarded the UGC Teaching Award by the University Grants Committee, Hong Kong.

Students from the aforementioned courses participated in an initiative entitled “Project *WeCan*,” launched in 2011 by the Wharf (Holdings) Limited (the Group). This business–university–community partnership attempted to provide disadvantaged local secondary school students with learning opportunities that would empower and prepare them for future educational or career endeavors through different programs, including service-learning programs provided by university students. During each academic year, students begin their SL course by learning about concepts and theories on topics such as positive youth development and service leadership. Equipped with the acquired knowledge and skills, students then communicate with community partners to understand the needs and interests of service recipients, based on which service activities are carefully tailored to meet the needs of the potential service recipients [20,21]. Since its inception, evaluation studies using both quantitative and qualitative methodologies have provided solid evidence that student services were professionally implemented, and the target recipients enjoyed and gained from these activities [21].

### 1.4. “WeCan” or “WeCan’t” under COVID-19?

The outbreak of COVID-19 did not waver the Initiative’s missions nor flicker the spirits of the students-PolyU collaborated with 11 local government-aided secondary schools throughout the 2019–20 academic year. First and foremost, lectures were delivered in real-time online via the Blackboard Collaborate Ultra video conferencing tool. Second, educators, schools, and students worked together to brainstorm and evaluate the feasibility and value-added of the indirect services proposed by students. Some students also used WhatsApp or called the service recipients to gain a better understanding of their needs and concerns. Appendix A outlines the indirect service activities successfully delivered.

### 1.5. The Present Study

Previous research has provided support for the positive impacts of Project *WeCan* on student service providers’ learning outcomes in terms of civic engagement, cognitive competence, academic performance, and other psychosocial competencies at both intrapersonal and interpersonal levels [20,22]. However, data obtained from these studies were pre-COVID-19.

Undeniably, COVID-19 has posed unprecedented challenges to different stakeholders of the corporate-community-university partnership central to the Project [23]. The findings of the present study will shed light on the post-pandemic development of SL. Against this background, we argue that it is important to gain a better understanding of university students’ (1) developmental outcomes; (2) perceptions towards the course content, lecturer, course effectiveness, and perceived benefits; and (3) overall satisfaction with the SL course during this collective crisis.

With reference to objective outcome evaluation, we were interested in investigating students’ changes in different developmental outcomes upon completing the SL course. To answer this question, a pretest-posttest design (i.e., pre-experimental design) was adopted to compare changes across various domains to address the following:

Research Question 1: Did university students (i.e., service providers) show positive changes (indexed by cognitive-behavioral competencies, positive identity, positive youth development, service leadership qualities, and life satisfaction) after completing the SL courses under COVID-19? In line with previous findings [20], we hypothesized that university students would demonstrate positive changes after taking the SL courses (Hypothesis 1). There are also research findings showing that objective outcome evaluation findings are related to subjective outcome evaluation [24,25].

To triangulate the findings, subjective outcome evaluation was also conducted to gauge the levels of satisfaction of both university students (i.e., service providers) and secondary school students (i.e., service recipients). Using subjective outcome evaluation, we addressed the following:

Research Question 2: What are the perceptions of the university students (i.e., service providers) on the SL courses delivered under COVID-19? Similarly, based on existing literature [20], we expected that university students would have positive perceptions regarding the SL course (Hypothesis 2).

Besides gauging views from the service providers, we also examined the views of the service recipients (i.e., high school students), which have been rarely examined in the literature:

Research Question 3: What are the views of the secondary school students (i.e., service recipients) on the SL services in Project *Wiccan* delivered under COVID-19? Consistent with previous findings [21], we predicted that the majority of the service recipients would perceive the Project in a positive manner (Hypothesis 3).

## 2. Materials and Methods

### 2.1. Objective Outcome Evaluation of University Students (Service Providers)

#### 2.1.1. Participants

A total of 201 full-time undergraduate students from various faculties and departments within PolyU enrolled in the two SL courses aimed at promoting positive youth development competencies and service leadership qualities throughout semesters 1 and 2 of the 2019–20 academic year. To match the pre- and post-test data, each participant had to provide a unique student identification number. The matching procedure yielded 124 sets of completed questionnaires for analyses, which obtained a 62% completion rate. The sample at the time of pre-test comprised 65 males (52.4%) and 59 females (47.6%). All participants were adults aged from 18–25 years old (M = 20.6; SD = 1.4). Students gave informed consent at the beginning of the term.

#### 2.1.2. Procedures

During the beginning of the first semester (i.e., weeks 1 to 3) of the 2019/20 academic year, students were invited to complete a self-report online questionnaire (i.e., the pre-test) via the Blackboard learning management system [20]. Upon the completion of all instructional and service provisional activities, students were once again invited to complete the post-test online. Ethical approval was duly obtained from the Human Subjects Ethics Committee of the University for the SL subjects. Prior to the commencement of data collection, the objectives of the study were explained to students. The voluntary nature of the research was highlighted. Should students wish to withdraw from the study at any time, penalties will not be applied. Finally, the confidentiality was reiterated; only designated members of the research team have access to the collected anonymous data. Student consent was sought prior to the commencement of the questionnaires. Participants were given ample time for completion.

#### 2.1.3. Instruments

Objective Outcome Evaluation Form. The online questionnaire administered aims to investigate the impacts of students’ SL experiences during the pandemic. Specifically, we measured changes in 6 domains [20], including (1) Cognitive-behavioral competencies; (2) Positive identity; (3) General positive youth development qualities; (4) Overall positive youth development; and (5) Service leadership qualities; and (6) Life satisfaction (see Table 1). Respondents indicated their agreement to items on a Likert-scale anchored from 1 = Strongly Disagree to 6 = Strongly Agree. The selected scales and subscales are reliable and valid, as shown in numerous existing research. Reliability analyses revealed Cronbach’s alphas of good internal consistency in the present study as well (see Table 2).

Positive Youth Development Attributes. The Chinese Positive Youth Development Scale (CPYDS) [26] was administered to assess students’ positive youth development attributes. A total of 31 core items from 10 positive youth development subscales were selected with relevance to the intended learning outcomes of the SL courses of interest in the present study. A total positive youth development score was also computed for each participant based on their scores from respective subscales. With reference to Shek and Ma’s [27] higher-order confirmatory factor analysis results, measures from three second-order factors, namely cognitive-behavioral competencies, positive identity, and general positive youth development qualities, were also used. The validity of the scale has been examined and demonstrated previously [26].

Life Satisfaction. The Satisfaction with Life Scale (SWLS) [28] consisting of 5 items was employed to gauge participants’ subjective well-being. Scores ranged from 5–30. Higher levels of life satisfaction were reflected by higher scores reported. A Chinese version of the scale widely used in Chinese contexts was administered [29].

Service Leadership Qualities. To assess students’ service leadership attributes, four subscales with a total of 34 items were included in the questionnaire. The “character strengths” subscale consists of 15 items tapping on whether respondents possess positive psychological assets, such as gratitude, justice, and perseverance. The 5-item “self-leadership” subscale examines students’ initiative when planning and managing their lives, as well as understanding oneself, which is crucial for self-development. As having a “caring disposition” is one of the main qualities of an effective service leader, eight items were included in the questionnaire to gain a better understanding of whether students are caring towards others. Finally, to succeed as a service leader, one must buy into its underlying “service leadership beliefs and values”. Thus, six items were incorporated in the questionnaire to assess whether students’ beliefs and values aligned with those proposed by the Service Leadership and Management Model. Items were adapted from the Service Leadership Attitude Scale, which has demonstrated high reliability and validity [30].

### 2.2. Subjective Outcome Evaluation for University Students (Service Providers)

#### 2.2.1. Participants and Procedures

Among the 201 university students enrolled in the two credit-bearing SL courses, 192 students completed the subjective outcome evaluation form, with a completion rate of 95.5%. At the end of the final semester of the 2019–20 academic year, university students completed a 41-item questionnaire. Ethical procedures were identical to those reported in the objective outcome evaluation above.

#### 2.2.2. Instrument

The subjective outcome evaluation form consisting of 38-items was administered to gauge university students’ perceptions on three domains: (1) Course content (10 items, α = 0.95); (2) the lecturer (10 items, α = 0.97); and (3) benefits of the subject (18 items, α = 0.98). For the first two domains, students were asked to rate their levels of agreement to the items ranging from 1 = Strongly Disagree to 5 = Strongly Agree. For the perceived benefits of the subject, responses were anchored from 1 = Unhelpful to 5 = Very Helpful. Higher scores indicate higher levels of satisfaction. Different versions of this measure have shown excellent psychometric properties [20].

### 2.3. Subjective Outcome Evaluation for Secondary School Students (Service Recipients)

#### 2.3.1. Participants and Procedures

Upon the completion of all services, we invited high school students from the five local government-aided secondary schools to complete a questionnaire. A total of 56 completed questionnaires were received and analyzed. Given that some secondary school students were aged less than 16 years old, parental consent was obtained. Ethical procedures, especially respondents’ rights, were clearly emphasized. Students were given ample time to complete the subjective outcome evaluation form.

#### 2.3.2. Instrument

A 28-item subjective outcome evaluation form was distributed to assess secondary school students’ (i.e., the service recipients’) perceptions towards: (1) the activities (9 items, α = 0.98); (2) PolyU students (i.e., the service providers) (9 items, α = 0.98); and (3) effectiveness of the activities (10 items, α = 0.99). This measure was found to be both valid and reliable [21]. The questionnaire has been administered to evaluate other service-learning courses in Chinese contexts [31]. Both sections on activities and PolyU students were rated on a 6-point Likert scale anchored at 1 = Strongly Disagree to 6 = Strongly Agree. Levels of effectiveness ranged from 1 = Very Unhelpful to 6 = Very Helpful. Higher scores indicate more positive perceptions.

## 3. Results

### 3.1. Objective Outcome Evaluation of University Students (Service Providers)

Analyses were carried out using the SPSS version 27.0 [32]. To begin with, reliability analyses were conducted on subscales administered at both pre- and post-test. Cronbach’s alpha coefficients for the subscales at both times are shown in Table 2. A high internal consistency across the majority of the scales was observed. The means and standard deviations of outcomes at both pre- and post-test are detailed in Table 2.

Research Question 1. To examine whether engagement in SL during COVID-19 instilled changes in students’ cognitive-behavioral competencies, positive identity, general positive youth development qualities, overall positive youth development indexed by the total score, service leadership qualities, and life satisfaction, a series of repeated-measure multivariate general linear model (GLM) analyses were conducted. They were subsequently followed by conducting repeated-measure univariate analyses of variance on respective outcomes.

As predicted, students demonstrated significant positive changes after taking part in the SL course. In terms of cognitive-behavioral competencies, there was a significant main effect of time, Wilk’s λ = 0.82, *F*(1, 109) = 23.67, *p* < 0.001, η^2^_p_ = 0.178. Further analyses revealed that students’ levels of self-determination (*p* < 0.01), behavioral competence (*p* < 0.001), and cognitive competence (*p* < 0.001) significantly increased at post-test.

Upon completing the SL course, students’ positive identity also improved, Wilk’s λ = 0.87, *F*(1, 119) = 17.18, *p* < 0.001, η^2^_p_ = 0.126. Specifically, students reported having a clearer and more positive identity (*p* < 0.001) and a more hopeful belief in what the future beholds (*p* < 0.05).

Adopting a positive youth development approach in SL even in unprecedented times was shown to be effective as evidenced by the significant changes in general positive youth development qualities of students at post-test, Wilk’s λ = 0.86, *F*(1, 104) = 16.67, *p* < 0.001, η^2^_p_ = 0.138. However, only levels of social competence and emotional competence were enhanced (*p*s < 0.001). Resilience, moral competence, and spirituality failed to yield significant positive changes. Despite the non-significant changes noted across several particular positive youth development constructs; taken as a whole, overall positive youth development qualities resulted in encouraging significant outcomes, Wilk’s λ = 0.81, *F*(1, 99) = 23.31, *p* < 0.001, η^2^_p_ = 0.184.

For service leadership qualities, a significant change at the multivariate level Wilk’s λ = 0.86, *F*(1, 51) = 8.53, *p* < 0.01, η^2^_p_ = 0.143 over time was observed. As to different attributes of effective service leaders, there was an increase in all the service leadership qualities after student participation in the SL course, including character strengths (*p* < 0.001), self-leadership, caring disposition, and service leadership beliefs and values (*p*s < 0.01).

Most importantly, in spite of the turbulent times, students’ life satisfaction improved significantly compared to before they took the SL course, Wilk’s λ = 0.94, *F*(1, 116) = 7.71, *p* < 0.01, η^2^_p_ = 0.062.

In summary, findings from the objective outcome evaluation supported our hypothesis (Hypothesis 1) that university students who completed the SL course demonstrated positive changes in their positive youth development and service leadership competencies, as well as their life satisfaction.

### 3.2. Subjective Outcome Evaluation of University Students (Service Providers)

Research Question 2. Descriptive statistics based on percentage analyses were performed to examine university students’ (i.e., service providers’) perceptions of their experiences. The percentage responses of service recipients who rated the items positively with ratings of either 4 = Agree or 5 = Strongly Agree (as detailed in Table 3).

With regards to the subject content, over 90% of students reported that the curriculum had clear objectives (91.6%). Over 80% of the respondents were also satisfied with the curriculum (83.2%), activities (86.4%), and pleasant atmosphere (89.0%).

Across all items tapping on university students’ perceptions towards their lecturers, over 90% of respondents agreed that the lecturer readily offered help in times of need (96.4%), was very involved (95.3%), and showed good professional attitudes (95.3%).

Taken as a whole, university students reported that the subject had brought benefits to them in different areas. For instance, the SL subject has helped nurture care and compassion (93.2%) and enabled them to appreciate roles that task competencies, character strength, and care play in effective leadership (91.6%), it also enhanced their social competence (90.6%). Fewer students reported benefits in the subject helping them explore the meaning for (71.4%) and love of (73.8%) life, and in facing the future with a positive attitude (79.5%), but the percentage figures are not low. Evidence gathered from the subjective outcome evaluation supported our hypothesis (Hypothesis 2) that service providers were highly satisfied with the SL course.

### 3.3. Subjective Outcome Evaluation of Secondary School Students (Service Recipients)

Research Question 3. In order to gain a better understanding of whether the needs of service recipients were met, and the benefits they have reaped from the various service activities, a series of descriptive statistics were conducted. A response rating that was above 4 was considered positive (see Table 4).

Taken as a whole, students were highly satisfied with the service activities, as evidenced by positive ratings of over 80% across items. Specifically, students reported active participation (85.7%) and found the activities to be appropriate (83.9%) and the atmosphere to be pleasant (83.9%). Service recipients were particularly satisfied with their service providers. University students were perceived to be well prepared (91.1%) and actively involved (91.1%). They also demonstrated professional attitudes (89.3%) and encouraged service recipients to participate in the various activities (89.3%).

Data also revealed that the secondary school students found SL under Project *WeCan* to be useful to them (85.2%). Some specific elements they found helpful included the development of positive relationships with adults in their lives (83.3%) and facing the future with a positive attitude (83.3%). As compared to other items, they reported slightly lower levels of satisfaction in terms of the program’s ability to help broaden their horizon (77.4%) and to enhance their interests towards study (75.9%). To sum up, the results gathered from the subjective outcome evaluation revealed service recipients’ high levels of satisfaction with the SL experience, which supported our final hypothesis (Hypothesis 3).

## 4. Discussion

The detection and rapid spread of COVID-19 led governments worldwide to swiftly implement measures (e.g., school closure, social distancing, lockdown) to reduce transmission of the disease. This transition meant taking lectures and student activities from face-to-face mode to online teaching and learning. This acculturation of pedagogical modalities at such a large-scale “might have produced the largest unplanned educational experiment ever undertaken” [33].

This study shed insight into this “unplanned educational experiment” in relation to SL and is unique for several reasons. First, a comprehensive literature search revealed a dearth of studies that investigated the impact of tertiary students’ learning experience under COVID-19. Second, among the studies that were yielded from the literature search, few of them focused particularly on courses like SL which comprises a strong experiential component. Third, while existing studies were conducted in a Western context, little is known about COVID-19′s impact on Chinese students and service recipients. Fourth, few of these studies have utilized validated assessment tools akin to the present study. Finally, to gain a more panoramic view of the pandemics’ impact on all stakeholders involved in a distinctive course like SL, perceptions of both service providers and recipients were gauged.

### 4.1. Objective Outcome Evaluation of University Students

Generally speaking, data gathered from the objective outcome evaluation tool revealed that university students (i.e., service providers) showed positive changes in cognitive-behavioral competencies, positive identity, positive youth development competencies, service leadership attributes, as well as life satisfaction after taking the SL course in spite of its online/offsite mode of delivery. The present findings are consistent with Lin and Shek’s [18] research which found that regardless of the mode of delivery (i.e., face-to-face vs. online), students acquired a wide range of positive youth development outcomes, service leadership qualities, and subjective well-being after completion of an SL course. Furthermore, Guthrie and McCracken [34] conducted a case study of an e-SL course and concluded that “the overall teaching approach serves multiple pedagogical functions focusing on the integration of experience and reflection as the means to further both academic growth and skill development” (p. 250). Research from developmental science has also demonstrated that structured out-of-school programs are effective in the promotion of positive youth development, as they entail elements including challenge, autonomy, and proactiveness [35]. It is noteworthy that there were non-significant changes in university students’ (service providers) levels of resilience, moral competence, and spirituality.

At first glance, the fact that the level of resilience did not change among university students after such an impactful academic year of SL may seem counterintuitive. However, it is the very unprecedented nature of COVID-19 per se that influenced the unpredictable outcome. Unlike most crises the population has been confronted with (e.g., climate change, terrorism, global famines, and natural disasters) where individuals within a community join forces physically and socially with the common goal and purpose to alleviate the consequences or rebuild what was destroyed, COVID-19-related policies encourage “separateness” for survival. Yet, the instinctual elements of social bonding, collectiveness, and deep human interconnectedness play imperative roles in the development of resilience [36]. It is plausible that the lack of social elements in person during their SL experience may have compromised students’ development of resilience.

In addition, it is not always the case that resilience can be nurtured after traumatic experiences; the nature and dose exposure of the threat must also be considered. In circumstances where individuals are confronted with events that threaten their basic security or livelihood, or when the threat is too novel and exceptionally fast—it is then “when resilience fails” [37]. Students may have been overwhelmed by the external impacts that required flexibility and adaptability to great changes. Some may have lacked the response capabilities or simply the time to digest, reflect, and internalize what is happening around them, making it difficult to gain a positive change in resilience through SL, especially when the pandemic is still ongoing. Longitudinal studies post-COVID-19 may otherwise reveal a significant positive change in resilience.

Zhao et al. [38] examined public moral motivation during the pandemic by analyzing posts on Chinese social media. Findings from their big data analysis revealed that the level of severity of the disaster influenced individuals’ moral motivation. Moral motivations increased as the disease got more serious. However, such effects faded quite rapidly once the disaster was under control and recovery commenced. Based on their findings, it is no longer surprising that university students’ levels of moral competence did not differ significantly, given that by the end of the academic year, policies were well implemented, and the disease transmission was under control. Also, the collected data revealed that university students had high levels of moral competence to begin with.

Connecting spirituality to SL in higher education is important as it allows students the opportunity to find their callings through the experience of serving the needy while also gaining discipline-specific knowledge for credit [39]. However, respondents from the present study did not report any changes in levels of spirituality after taking SL under the pandemic. This seems to contradict existing findings which provided evidence that school-based socio-emotional learning programs which incorporate social service activities are effective in nurturing students’ spirituality.

Often, the development of spirituality is conceptualized as an individual’s search for meaning in life and self-transcendence. However, to develop self-transcendence, youths need to experience and explore one’s “self” as embedded in a larger and collective context. Therefore, one must not overlook the social and relational realms of spiritual development [40]. In fact, spiritual development may also be conceptualized as a communal process. Along with this rationale, Revell [41] highlighted the importance of fostering a sense of belongingness and civic-mindedness within schools and the community.

During COVID-19, however, universities were mandated to be closed, which denied students the opportunity to form strong social bonds with their peers and teachers. Camaraderie was grossly limited in virtual classes [42]. Moreover, they were also unable to deliver service to the needy within the community in-person, all of which may have taken a toll on their development of spirituality. Indeed, the search for one’s purpose and meaning in life within the community without “human contact” can be challenging for some. The development of future e-SL courses should consider ways to further enhance students’ sense of belongingness and guide them to explore one’s “self” in virtually construed social environments/communities.

As it can be seen, practicing e-SL has its drawbacks. It has been argued that personal interactions between teachers and students, and among students themselves, are vital parts of the educational experience but are often limited in online classes. E-Learning also requires strong motivation and time management skills from learners. Clarifications and explorations of vague concepts and ideas may transmit more effectively in-person [13]. Therefore, we were also interested in examining how service providers and recipients who participated in the Project *WeCan* perceived the experience during the COVID-19 pandemic.

### 4.2. Subjective Outcome Evaluations

Data gathered from the subjective outcome evaluations from both university students (i.e., service providers) and secondary school students (i.e., service recipients) were positive. University students were highly content with the course content, lecturer, and perceived benefits of the e-SL course. Our encouraging findings are consistent with a nationwide study coordinated by the Chinese Ministry of Education of over 39,000 university students who took online courses in China during the COVID-19 outbreak. A vast majority of participants perceived that the intended learning outcomes of courses were fully attained. They also commended their teachers for bringing positive energy and enthusiasm to help them alleviate distress from quarantine. Prior studies have also outlined the advantages of e-Learning, including the increase in peer-to-peer communication, flexibility in teaching and learning, and availability of lecture videos and materials at all times accounting for individual learning differences or styles [13].

From the perspective of service recipients from the secondary schools in Hong Kong, they were also highly contented with the service activities that the university students designed and implemented. They admired the professionalism and encouraging attitudes of the service providers. The service recipients also claimed that the wide variety of indirect service activities delivered to them were effective for their academic and personal growth (see Appendix A for details of the indirect service delivered). The positive feedback we received add to existing literature detailing successful attempts of shifting focus from direct SL to indirect, research- and advocacy-based service projects during the pandemic [43].

### 4.3. Theoretical and Practical Implications

Various implications are derived from our findings. Traditional frameworks of SL were initially developed based on psychological notions such as Contact Theory under the assumption that face-to-face contact would enhance personal and social understanding between members of different groups within the community [44]. Rapid technological advancements have led to the emergence of e-Learning and hybrid learning in the educational sector. Recently, due to the outbreak of COVID-19, e-Learning has become a necessity for the continuous delivery of curricula in most countries. Yet, little is known about the effectiveness of e-Learning when applied to unique experiential courses such as SL. Previous offerings of the two SL courses under Project *WeCan*, prior to the pandemic, have been well-received by different stakeholders [22]. The present study coincides with those findings and contributes theoretically as a testament of the successful shift from a traditional face-to-face mode of SL to an extreme mode of online knowledge and service delivery.

Our findings also shed light on the learning outcomes and goals of SL. Shek [45] analyzed the protests in Hong Kong, just prior to the COVID-19 outbreak, from a quality-of-life perspective. Some pre-existing “fuels” that have led up to the protest were identified, including a weak identification with the Chinese national identity, lack of upward mobility, lack of life skills education for adolescents, and the unsystematic and uncoordinated civic and national education in Hong Kong. In the US, Saltmarsh [46] noted that “as a curricular outcome in courses across the disciplines, civic learning remains largely unaddressed” (p. 52).

Our results from both the objective and subjective outcome evaluations point to the future development of SL theories and models to incorporate e-Learning elements in the nurturance of positive youth development competencies, life skills, soft skills, and civic values of our youths [18]. Finally, research on the pedagogical and psychological underpinnings of effective implementation of e-SL is still needed. These may include the social presence of respective stakeholders, affective responses to one another, respect, shared values, trust, feedback, cohesiveness, and self-efficacy [47].

In terms of practical implications, the “forced limitations” of e-SL led to practical advancements. First, in terms of planning, students had to think out of the box and utilize their cognitive competence to develop creative service activities—some that may have never crossed their minds before. Service providers had to use various channels (e.g., email, online conferencing, and telephone) to gauge the needs of their clients, which required qualities and skills such as care, empathy, and active listening. In terms of implementation, both university and secondary school students’ interpersonal and communication skills were also sharpened. In terms of reflection, for many students, this may have been one of the greatest global crises that they have witnessed and experienced in their life, which encourages them to reflect, be grateful for, and enhance their psychological flexibility to maintain a positive quality of life [48]. Yet, all these require psychosocial competencies in the students which may be lacking in Hong Kong [49]. Looking forward, this shift in landscape may impact SL even after COVID-19, allowing for the forging of even stronger university-community partnerships and the exploration of new ways to restructure and reimagine the entire SL experience for all parties. The flexible and sustainable SL model reported can be applied to other parts of the world in response to future public health emergencies. With the improved technological literacy of many, non-face-to-face service may also be delivered to the needy in remote places without physical and geographical constraints (i.e., given stable access to the Internet).

### 4.4. Limitations and Future Directions

While the present study yielded both theoretical and practical implications for e-SL, there are some limitations. First, the study had a limited sample size which may compromise its generalizability. However, despite this, robust effect sizes were observed. Second, only students from two SL courses under Project *WeCan* were surveyed. Third, we only sought the views of university and secondary school students who were involved in the Project through self-report. While the use of self-report is widely supported in social science and evaluation research [50], Day and colleagues [33] suggested that “well-designed, multi-institutional, quantitative studies with large samples” (p. 10) can shed light on the impacts of remote teaching and learning. As such, future investigations may benefit from a larger, more heterogeneous sample, to include students from a wider range of academic disciplines taking various SL courses and to gather the perceptions of different stakeholders, including principals, teachers, social workers, or parents, regarding participants’ gains and their views for a more holistic picture of e-SL. Fourth, our study adopted a pretest-posttest design in examining the immediate changes and perceptions of participants upon completion of their SL course. In the future, quasi-experimental design with a control group should be carried out [51,52]. Follow-up studies may also be conducted to examine the sustained effects on participants’ personal or professional growth. Finally, it would be theoretically significant to examine how the positive outcomes of SL (e.g., positive youth development attributes) may further impact the well-being of the service recipients [53,54].

## 5. Conclusions

To conclude, findings from the present study support e-SL as an effective pedagogical approach critical to the promotion of positive youth development competencies, service leadership qualities, and life satisfaction amongst adolescents even during difficult times where youth ecological systems are in turmoil. In conjunction with the widespread benefits reaped from the extreme e-SL model (i.e., having online lecture delivery and indirect service provision) demonstrate its versatility in incorporating technology and adaptability to address remote needs and future emergencies.

## Figures and Tables

**Table 1:** Description of Objective Outcome Evaluation Form for University Students (Service Providers).

	No. of Items	Sample Items
Cognitive-behavioral competencies (3 factors)		
Self-determination	3	I am confident about my decisions.
Behavioral competence	2	I can face criticism with an open mind.
Cognitive competence	4	I try new ways to solve my problems.
Positive identity (2 factors)		
Clear and positive identity	2	I am a person with self-confidence.
Beliefs in the future	3	I have confidence to solve my future problems.
General positive youth development qualities (5 factors)		
Resilience	3	When I face difficulties, I do not give up easily.
Social competence	3	I know how to communicate with others.
Moral competence	4	I have high moral standards about my behaviors.
Emotional competence	3	When I have conflict with others, I can usually manage my emotions.
Spirituality	4	I have found my purpose in life.
Service leadership qualities		
Character strengths	15	I am grateful for many things in my life.
Self-leadership	5	I have a good planning of my life.
Caring disposition	8	I am always ready to lend a hand to those in need.
Service leadership beliefs and values	6	Leadership is a service for self, others, groups and the society.
Life satisfaction	5	In most ways, my life is close to my ideal.

**Table 2:** Outcome changes between pre-test and post-test from the Objective Outcome Evaluation Form by university students (service providers) (*n* = 124).

	Pre-Test		Post-Test		*F*	η^2^_p_
	Mean (SD)	α	Mean (SD)	α		
Cognitive-behavioral competencies					23.67 ***	0.178
Self-determination	4.57 (0.79)	0.87	4.73 (0.76)	0.83	7.18 **	0.057
Behavioral competence	4.58 (0.78)	0.77	4.94 (0.76)	0.83	29.48 ***	0.196
Cognitive competence	4.66 (0.70)	0.85	4.96 (0.70)	0.91	30.80 ***	0.210
Positive identity					17.18 ***	0.126
Clear and positive identity	4.09 (1.00)	0.84	4.40 (1.06)	0.90	23.80 ***	0.164
Beliefs in the future	4.77 (0.75)	0.80	4.89 (0.83)	0.84	3.91 *	0.031
General positive youth development qualities					16.67 ***	0.138
Resilience	4.58 (0.89)	0.87	4.69 (0.93)	0.93	3.07	0.026
Social competence	4.73 (0.75)	0.91	4.98 (0.79)	0.95	25.52 ***	0.174
Moral competence	4.68 (0.57)	0.40	4.70 (0.62)	0.31	0.168	0.001
Emotional competence	4.61 (0.75)	0.80	4.89 (0.83)	0.88	21.61 ***	0.149
Spirituality	4.25 (0.80)	0.63	4.33 (0.82)	0.52	1.77	0.015
Total positive youth development					22.31 ***	0.184
Service leadership qualities					8.53 **	0.143
Character strengths	4.61 (0.57)	0.90	4.84 (0.62)	0.92	21.00 ***	0.165
Self-leadership	4.56 (0.70)	0.88	4.79 (0.70)	0.90	11.09 **	0.087
Caring disposition	4.86 (0.64)	0.94	5.02 (0.63)	0.94	9.06 **	0.074
Service leadership beliefs and values	4.85 (0.65)	0.92	5.07 (0.66)	0.95	8.30 **	0.115
Life satisfaction	3.90 (1.06)	0.91	4.14 (1.15)	0.94	7.71 **	0.062

*** *p* < 0.001; ** *p* < 0.01, * *p* < 0.05.

**Table 3:** Descriptive statistics and positive responses from the Subjective Outcome Evaluation by university students (service providers) (*n* = 192).

			Positive Responses (4–5 Ratings)	
Item		Mean (SD)	*n*	%
Subject Content ^a^				
1	The objectives of the curriculum are very clear.	4.25 (0.63)	176	92.6
2	The design of the curriculum is very good.	4.06 (0.82)	159	83.2
3	The activities were carefully arranged.	4.12 (0.85)	165	86.4
4	The classroom atmosphere was very pleasant.	4.26 (0.73)	170	89.0
5	There was much peer interaction amongst the students during classes.	4.18 (0.70)	166	87.4
6	I participated actively during lessons (including discussions, sharing, games, etc.).	4.19 (0.68)	186	87.5
7	I was encouraged to do my best.	4.26 (0.68)	175	91.6
8	The learning experience I encountered enhance my interest towards the subject area.	4.12 (0.82)	158	83.2
9	Overall speaking, I have very positive evaluation of the program.	4.13 (0.84)	165	86.4
10	On the whole, I like this curriculum very much.	4.00 (0.91)	151	79.1
Lecturer ^a^				
11	The lecturer(s) had a good mastery of the curriculum.	4.35 (0.71)	177	92.2
12	The lecturer(s) was (were) well prepared for the lessons.	4.42 (0.67)	179	93.2
13	The teaching skills of the lecturer(s) were good.	4.42 (0.65)	182	94.8
14	The lecturer(s) showed good professional attitudes.	4.47 (0.66)	183	95.3
15	The lecturer(s) was (were) very involved.	4.48 (0.65)	183	95.3
16	The lecturer(s) encouraged students to participate in the activities.	4.44 (0.62)	180	94.2
17	The lecturer(s) cared for the students.	4.48 (0.66)	179	93.2
18	The lecturer(s) was (were) ready to offer help to students when needed.	4.53 (0.61)	185	96.4
19	The lecturer(s) had much interaction with the students.	4.42 (0.69)	177	92.7
20	Overall speaking, I have very positive evaluation of the lecturer(s).	4.48 (0.61)	183	95.3
Perceived benefits ^b^				
21	It has enhanced my social competence.	4.20 (0.77)	173	90.6
22	It has improved my ability in expressing and handling my emotions.	4.07 (0.83)	159	83.7
23	It has enhanced my critical thinking.	4.06 (0.87)	158	82.7
24	It has increased my competence in making sensible and wise choices.	4.07 (0.80)	161	84.7
25	It has helped me make ethical decisions.	4.18 (0.76)	171	89.1
26	It has strengthened my resilience in adverse conditions.	4.14 (0.83)	161	84.3
27	It has strengthened my self-confidence.	4.08 (0.89)	159	83.2
28	It has helped me face the future with a positive attitude.	3.97 (0.93)	151	79.5
29	It has enhanced my love for life.	3.84 (1.02)	141	73.8
30	It has helped me explore the meaning of life.	3.81 (1.03)	137	71.4
31	It has enhanced my ability of self-leadership.	4.11 (0.78)	162	84.4
32	It has helped me cultivate compassion and care for others.	4.22 (0.70)	179	93.2
33	It has helped me enhance my character strengths comprehensively.	4.09 (0.79)	166	86.5
34	It has enabled me to understand the importance of situational task competencies, character strength and caring disposition in successful leadership.	4.19 (0.69)	174	91.6
35	It has promoted my sense of responsibility in serving the society.	4.13 (0.83)	166	86.5
36	It has promoted my overall development.	4.08 (0.83)	163	85.8
37	The theories, research and concepts covered in the course have enabled me to understand the characteristics of successful service leaders.	4.16 (0.73)	171	89.5
38	The theories, research and concepts covered in the course have helped me synthesize the characteristics of successful service leaders.	4.09 (0.73)	163	85.3

Note: ^a^ items were rated on a 5-point Likert-type scale with 1 = strongly disagree, 2 = disagree, 3 = neutral, 4 = agree, 5 = strongly agree. ^b^ items were rated on a 5-point Likert-type scale with 1 = unhelpful, 2 = not very helpful, 3 = not sure, 4 = helpful, 5 = very helpful.

**Table 4:** Descriptive statistics and positive responses from the Subjective Outcome Evaluation by secondary school students (service recipients) (*n* = 56).

			Positive Responses (4–6 Ratings)	
Item		Mean (SD)	*n*	%
Service Activities ^a^				
1	The content design of the activity is very good.	4.50 (1.14)	45	80.4
2	The format of the activity is appropriate.	4.59 (1.16)	47	83.9
3	The atmosphere of the activity was pleasant.	4.70 (1.22)	47	83.9
4	There was much peer interaction amongst the students.	4.45 (1.19)	45	80.4
5	I participated in the activity actively.	4.59 (1.16)	48	85.7
6	I was encouraged to do my best.	4.46 (1.38)	46	82.1
7	The learning experience enhanced my interests towards the service.	4.57 (1.36)	46	82.1
8	Overall speaking, I have a very positive evaluation on the activity.	4.66 (1.27)	46	82.1
9	On the whole, I like this activity very much.	4.57 (1.29)	47	83.9
Service providers ^a^				
10	PolyU student(s) was (were) well prepared for the activity.	4.82 (1.05)	51	91.1
11	PolyU student(s) showed professional attitudes.	4.79 (1.06)	50	89.3
12	PolyU student(s) understood my needs and potentials.	4.55 (1.11)	45	80.4
13	PolyU student(s) was (were) actively involved.	4.86 (1.02)	51	91.1
14	PolyU student(s) encouraged me to participate in the activity.	4.79 (.99)	50	89.3
15	PolyU student(s) cared about me.	4.77 (1.09)	48	85.7
16	PolyU student(s) showed readiness to offer help to me when needed.	4.86 (1.16)	49	87.5
17	PolyU student(s) had much interaction with us.	4.66 (1.16)	47	83.9
18	Overall speaking, I have a very positive evaluation on the PolyU students.	4.91 (1.01)	51	91.1
Perceived effectiveness of service activities ^b^				
19	It has strengthened my resilience in adverse conditions.	4.55 (1.27)	45	81.8
20	It has helped me to face the future with a positive attitude.	4.61 (1.24)	45	83.3
21	It has enhanced my self-confidence.	4.51 (1.28)	43	81.1
22	It has broadened my horizon.	4.53 (1.32)	41	77.4
23	It has enhanced my interests towards study.	4.57 (1.23)	44	75.9
24	It has enhanced my ability of caring.	4.60 (1.29)	43	81.1
25	It has helped me to develop a good relationship with adults (for example, teachers, parents, etc.)	4.53 (1.32)	45	83.3
26	It has promoted my aspiration.	4.52 (1.37)	43	81.1
27	It has promoted my holistic development.	4.58 (1.37)	43	81.1
28	On the whole, I think the activity is very useful to me.	4.69 (1.26)	46	85.2

Note: ^a^ items were rated on a 6-point Likert-type scale with 1 = strongly disagree, 2 = disagree, 3 = slightly disagree, 4 = slightly agree, 5 = agree, 6 = strongly agree. ^b^ items were rated on a 6-point Likert-type scale with 1 = very unhelpful, 2 = unhelpful, 3 = slightly unhelpful, 4 = slightly helpful, 5 = helpful, 6 = very helpful.

## Data Availability

The data presented in this study are available on request from the corresponding author. The data are not publicly available due to privacy.

## Appendix A

**Table A1:** Indirect SL activities and respective Project *WeCan* objectives.

Project *WeCan* Objectives	Examples of Indirect Service Deliverables Produced by SL Students
Enhance students’ communication skills	An e-Learning package for using Excel Visual Basic Applications
	The package included PowerPoint decks, walk-through videos, notes, exercises
	An English idiom learning package entitled “Funny English for Senior Form Students”
	The learning package included humorous videos, worksheets, lecture notes, posters, and booklets
	A range of videos helping students to prepare for their Diploma of Secondary Education (DSE) examinations
	Topics of the videos included useful information and study tips for taking Chinese and English oral or written examinations, as well as different elective subjects
	Real-time synchronous English workshops helping students to prepare for their public examinations
	Past papers were used as samples with a wide range of topics including e-sports, video games, healthy lifestyle, etc.
Increase students’ exposure	An Internet platform providing secondary school students with useful information on post-secondary study or career options
	Videos of university students sharing experiences from previous international exchange and internship programs
Enhance students’ basic competence	Educational videos imparting knowledge on hygiene and public health
	Real-life scenarios were incorporated dramatically to enhance resonance and solidify audiences’ memories
Empower students for pursuing higher studies and future careers	Real-time synchronous career talks introducing disciplines such as tourism, nursing, design accounting, aviation, etc.
	University students introduced the nature of different subject disciplines, entry requirements, career development pathways, etc.
	A mini-video series entitled “One Man One Story” sharing personal anecdotes of both struggles and victories on their life journey before joining The Hong Kong Polytechnic University
	Real-time synchronous mock interviews helping secondary students to prepare for their academic or career goals
	University students guided secondary school students to research and prepare application materials for post-secondary institutions or organizations of interest
Develop students’ common sense	A set of e-Tutorials teaching students interviewing skills for progressing academically (e.g., for associate degree candidates) or professionally (i.e., for those who wish to enter the workforce)
	The content of the tutorials included how to prepare a self-introduction, tips in responding to sample interview questions, interview etiquettes and mannerisms (e.g., Dos and Don’ts)
Cultivate students’ character	A personality test administered enhancing students’ self-understanding of one’s own strengths and weaknesses
	Students received immediate feedback online with explanations
